# Using transcranial direct current stimulation to improve outcomes and reduce hip osteoarthritis burden (the STIM HIPS study): A protocol for a randomised, triple blind controlled trial

**DOI:** 10.1016/j.jsampl.2024.100056

**Published:** 2024-04-10

**Authors:** Myles C. Murphy, Janet L. Taylor, Paola Chivers, Jonathan M. Hodgson, Casey Whife, Cobie Starcevich, Liam Tapsell, Joanne Kemp, Andrea Mosler

**Affiliations:** a Nutrition and Health Innovation Research Institute, School of Medical and Health Sciences, Edith Cowan University, Joondalup, Western Australia, Australia; b School of Health Sciences, The University of Notre Dame Australia, Fremantle, Western Australia, Australia; c School of Medical and Health Sciences, Edith Cowan University, Joondalup, Western Australia, Australia; d Institute for Health Research, The University of Notre Dame Australia, Fremantle, Western Australia, Australia; e SportsMed Subiaco, St John of God Health Care, Subiaco, Western Australia, Australia; f La Trobe Sport and Exercise Medicine Research Centre, La Trobe University, Bundoora, Victoria, Australia

**Keywords:** Hip, Osteoarthritis, Brain stimulation, tDCS, Cortical inhibition

## Abstract

**Background:**

Transcranial direct current stimulation (tDCS), *via* an electrical current being sent through the brains motor cortex, can elicit pain reduction and improved function in people with knee osteoarthritis (OA), compared to a sham. However, it is unknown whether tDCS-induced reductions in pain can be expected in hip OA given differences between hip and knee OA phenotypes.

**Methods:**

Two-armed (*n* ​= ​39 per arm), triple-blind, randomised controlled trial, with an 8-week intervention window and 8-week post-intervention follow-up assessing the efficacy of real anodal tDCS plus exercise versus sham tDCS plus exercise. Primary outcome measure is the International Hip Outcome Tool–33 (iHOT-33).

**Aims:**

The primary objective of this randomised controlled trial is to quantify the effect of tDCS and exercise on pain, disability and quality of life in people with hip OA. Our secondary objectives include: 1) quantifying the influence of motor cortex excitability and conditioned pain modulation on treatment effects, and 2) quantifying the economic cost/benefit of tDCS for improving health-related quality of life in people with hip OA.

**Analysis:**

Data distributions will be examined for each outcome and guide preliminary statistical between group test selections. Repeated mixed effects models will determine between-group differences for the primary outcome (iHOT-33), accounting for relevant confounders (i.e., age; sex; body mass index; radiographic severity) with relevant model assumptions examined. Secondary analysis will determine between-group differences for the other outcomes of interest (cortex excitability and conditioned pain modulation).

**Implications:**

This randomised controlled trial investigates a novel intervention to improve pain, function and quality of life in people with hip OA.

**Key points**

1. The transcranial direct current stimulation (tDCS) intervention described in this study protocol aims to improve pain and function in people with hip osteoarthritis. tDCS has been demonstrated to reduce pain, and by capitalising on the potential reduction of pain should also enable people to increase their physical activity levels. Providing the benefits of tDCS by relieving pain, plus the associated benefits of increased physical activity.
2. Transcranial direct current stimulation (tDCS) is hypothesised to improve hip-related pain and function *via* three mechanisms: 1) directly reducing pain through tDCS application, 2) increased physical activity levels due to reduced pain from tDCS application, and 3) enhanced outcomes from rehabilitation due to additional muscular adaptation by using tDCS to reduce cortical inhibition.

## 1 Background

Osteoarthritis (OA) is a worldwide problem [1] affecting more than 3 million Australians. OA causes significant personal burden with higher years lived with disability than type II diabetes, ischaemic heart disease or stroke [2]. OA also has a formidable impact on the workforce (>60,000 Australians with lost productive life years) and economic burden (hip OA replacement costs expected to hit $AUD953 million per annum by 2030) [3]. Hip OA has the largest increase in prevalence (171% from 1990 to 2019), compared to other joints [2]. However, research into the prevention, risk factors and management of hip OA is lacking [4], especially compared to knee OA. One field particularly under-researched in hip OA is neurophysiological drivers and treatments.

The brain acts like a car; an accelerator contracts a muscle, a brake stops it. A recent systematic review showed that individuals with hip and knee OA cannot ‘release the brake’ even when contracting at their fullest (maximal voluntary activation), they are unable to generate what should be maximal force [4]. Thus, large differences are apparent in voluntary activation between people with OA and healthy controls, as well as moderate differences between the symptomatic and asymptomatic limb in people with OA [4]. Furthermore, inhibition of the motor cortex has been associated with worse knee OA pain severity and disability [5]. Pilot studies in knee OA have shown brain stimulation techniques like transcranial direct current stimulation (tDCS) can improve pain, disability and function by up to 60% [6]. However, whilst tDCS produces meaningful improvements in knee OA pain [[6], [7], [8]], the effect of performing tDCS during exercise rehabilitation for hip OA is currently unknown.

A tDCS device sends an electrical current through the brain's motor cortex. This current makes it easier for the brain to drive the muscle to activate (i.e., release the ‘brake’), thus increasing muscle strength [9]. tDCS also influences pain. In controls, in the presence of noxious stimuli (e.g., injury), the body releases natural, endogenous analgesia to reduce sensitivity, termed conditioned pain modulation (CPM) [10]. However, in OA, the pain response is impaired and related to pain pattern as well as severity [11]. tDCS, *via* the electrical current being sent through the brain, can alter the CPM response in knee OA [12] and improve pain, compared to a sham [6]. However, it is unknown whether tDCS-induced reduction in pain can be expected in hip OA when voluntary activation deficits appear to be larger compared with knee OA [4].

### 1.1 Hypothesis

tDCS will meaningfully improve pain, disability, and quality of life (QoL) in people with hip OA.

### 1.2 Objectives

The primary objective of this randomised controlled trial is to quantify the effect of tDCS and exercise on pain, disability and QoL in people with hip OA. Our secondary objectives include: 1) quantifying the influence of motor cortex excitability and CPM on treatment effects, and 2) quantifying the economic cost/benefit of tDCS for improving health-related QoL in people with hip OA.

## 2 Methods

### 2.1 Study design

Two-armed, triple-blind, randomised controlled trial, with an 8-week intervention window and 8-week post-intervention follow-up. The study will be reported according to CONSORT guidelines [13], and include an economic analysis and process evaluation.

### 2.2 Clinical trial registration

The trial (including statistical plans) has been registered in the Australia New Zealand Clinical Trial Registry (ACTRN12624000129583p) and will be regularly updated.

### 2.3 Ethical considerations

Ethical approval has been granted by the Edith Cowan University Human Research Ethics Committee (ID: 2023-04901-MURPHY) and all participants will provide informed, electronic consent.

### 2.4 Funding information

This clinical trial is funded by a Raine Medical Research Foundation Priming Grant (G1006856), awarded to Dr Myles C Murphy.

### 2.5 Consumer engagement

Previous consumer input suggested that non-pharmacological interventions that reduce pain while exercising would be beneficial, and consumers supported the novel use of tDCS. Consumers also highlighted barriers, which informed study design (e.g., >3 in-person tDCS sessions per week was not feasible). A research buddy will be sourced in collaboration with the WA Health Translation Network Community and Consumer Involvement Program to provide input into the project as needed.

### 2.6 Participants

Recruitment of 78 consenting, adult participants (39 per arm) with a clinical and radiological diagnosis of hip OA will be undertaken (includes accounting for dropouts). The clinical and radiological diagnosis will be made *via* a Sports and Exercise Physician. The clinical diagnostic criteria include: activity-related hip pain (>3/10) and a positive Flexion Adduction and Internal Rotation (FADDIR) test. Radiological OA will be categorised as a Kellgren Lawrence score of greater than or equal to 2 [14].

Participants will be excluded for: rehabilitation in past 6-months; previous lower-limb surgery; hip joint injection within 3 months; neurological conditions; cardiometabolic conditions that preclude exercise; inability to commit to rehabilitation; specific transcranial magnetic stimulation, tDCS or CPM contraindications (Table 1); inability to understand English; <40 years of age.

**Table 1:** Contraindications to transcranial magnetic stimulation, transcranial direct current stimulation and conditioned pain modulation.

Assessment	Exclusion Criteria
Transcranial magnetic stimulation and transcranial direct current stimulation	Pregnancy
	Neurological conditions/illness, including epilepsy/convulsion/seizure
	Vascular, traumatic, tumorous, infectious, or metabolic lesion of the brain, even without history of seizure, and without anticonvulsant medication
	Previous or current implants in their body that may be triggered or heated by an electrical current (e.g. pacemaker, intracranial shunts, artificial cochlea, etc)
	Any mental implanted in their head (e.g. surgical clips, staples, shrapnel)
	Frequent or intense headaches
	Previous brain trauma or neurosurgical intervention
	Serious medical complications (e.g. advanced pulmonary, cardiac, liver or kidney disease)
Conditioned Pain Modulation	Cold urticaria
	Raynaud's phenomenon

### 2.7 Recruitment

Participants will be recruited over 14 months, *via* clinical networks, clinical research partners (e.g., orthopaedic clinics), social media (e.g., hip OA groups), and word-of-mouth. The research team have an extensive clinical network and expect recruitment to be highly feasible at 5 participants/month.

### 2.8 Randomisation and blinding

Following recruitment, participants will be randomised using a computer-generated randomisation list in RedCap® to one-of-two groups: 1) real anodal tDCS ​+ ​exercise or, 2) sham anodal tDCS ​+ ​exercise. Each tDCS device will be numbered (numbers ranging from 1 to 8 as we have 10 tDCS devices, allowing 2 in reserve if devices are lost/damaged). The study coordinator will program each tDCS device to a real or sham program [1:1 (real:sham)]. Participants will then be randomly allocated *via* the study coordinator to a device and will use this single device during all rehabilitation sessions. Thus, all assessors and clinicians performing assessments and rehabilitation will be blinded.

### 2.9 Intervention

Dr Murphy will train partner physiotherapists in delivering the tDCS and exercise-based interventions for STIM-Hips. The exercise rehabilitation intervention will be identical between groups and will be reported as per the Consensus on Exercise Reporting Template (CERT) [15] in Table 2. Participants will be provided an 8-week gym membership at the provider physiotherapy clinic. The intervention will involve 8 weeks of physiotherapist-supervised exercise, consisting of three in-person visits/week in which participants receive tDCS during each session. Exercise will be based on templates, sent *via* Physitrack®, however these will be individually adapted as able and progressed when participants can complete the prescribed exercise.

**Table 2:** Consensus on exercise reporting template.

Item	Protocol
Detailed description of the type of exercise equipment	The majority of exercises will use body weight resistance, however the following additional equipment will be utilised: knee extension machine, hamstring curl machine, leg press machine, circular TheraBand.
Detailed description of the qualifications, expertise and/or training	All exercises will be prescribed by a qualified physiotherapist, with greater than two years of experience in managing chronic hip pain.
Describe whether exercises are performed individually or in a group	Exercises will be prescribed individually and performed individually, however there may be other rehabilitation patients and clinicians in the rehabilitation gymnasium at the same time.
Describe whether exercises are supervised or unsupervised; how they are delivered	Participants will receive 1:1 supervision by a physiotherapist for one rehabilitation session per fortnight (which will include progressing exercises). All other rehabilitation sessions will be unsupervised.
Detailed description of how adherence to exercise is measured and reported	Attendance at the rehabilitation centre will be recorded *via* private practice standard booking system.
Detailed description of motivation strategies	Participants will receive reminder text messages about physiotherapy consultations.
Detailed description of the decision rule(s) for determining exercise progression	All exercises will be prescribed so they are either pain free or have minimal pain (≤2/10 on a numerical rating scale of pain) when performed. Exercises will be progressed when all sets and repetitions can be performed.
Detailed description of how the exercise program was progressed	Not applicable.
Detailed description of each exercise to enable replication	The exercise progressions will be based on patient function but must include: • A single joint exercise for each of the following muscle groups: lumbar extensors; abdominals; hip extensors; hip flexors, hip abductors; hip adductor; knee flexors; knee extensors; ankle plantar flexors. • A multi joint squat pattern (e.g., squat) and bend pattern (e.g., deadlift) movement. • One balance-based exercise (e.g., star excursion balance).
Detailed description of any home programme component	Not applicable.
Describe whether there are any non-exercise components	The additional intervention is provided below.
Describe the type and number of adverse events that occur during exercise	Adverse events will be reported to all study staff as they occur.
Describe the setting in which the exercises are performed	Exercises will be performed in a physiotherapy rehabilitation gymnasium.
Detailed description of the exercise intervention	Participants will be provided double leg single joint exercises (e.g., double leg bridge), before being progressed to single leg single joint exercises (e.g., single leg bridge) and then more highly loaded single leg single joint exercises (e.g., single leg hip thrust). Sets and repetitions will be based on endurance repetitions (e.g., 3 ​× ​15 reps) and performed three times per week.
Describe whether the exercises are generic (one size fits all) or tailored	Exercises will follow the description above but will be individually tailored to the participant and progressed as able.
Detailed description of how exercises are tailored to the individual	Exercises will be progressed when participants can perform 3 ​× ​15 repetitions or regressed if they are causing pain >2/10.
Describe the decision rule for determining the starting level	All participants will be commenced with double leg single joint exercises and progressed as able.
Describe how adherence or fidelity is assessed/measured	Adherence will be determined as the number of sessions attended across the 8-week intervention divided by the number of prescribed sessions.
Describe the extent to which the intervention was delivered as planned	Not applicable.

The tDCS devices (Brain Premier tDCS E1 Plus) will deliver a weak current [2 milliamps (mA)] from a 35 ​× ​35 ​mm anodal electrode [that increases cortical excitability [16]] on the scalp at the vertex (overlying primary motor cortex of the more symptomatic limb) to a cathodal electrode placed over the deltoid tubercle on the upper arm. The physiotherapist will also train the participant in how to self-perform tDCS on non-supervised days, and will be available to provide assistance if needed during non-supervised sessions. Other than electrical stimulation, real and sham procedures are identical. Real tDCS will be performed at 2 ​mA for 20 ​min whilst completing exercise rehabilitation. Sham tDCS will be applied during exercise with the placebo setting active, ramping to give the sensation of tDCS, then dropping to no output with the screen falsely reporting 2 ​mA of output.

### 2.10 Outcome measures

All baseline assessments will be completed within 7-days prior to commencing the intervention and follow-up will occur as per our assessment schedule (Table 3). Self-reported outcome measures will be recorded *via* RedCap® and will take 20–30 ​min for each participant. Physical assessment measures will be recorded by the principal investigator and research assistant directly into RedCap®. Physical assessments will occur at the Edith Cowan University neurophysiology laboratory, in accordance with existing standard operating procedures, and are expected to take ∼120 ​min.

**Table 3:** STIM-Hips study assessment schedule.

	Variable	Weeks			
		0	4	8 (post-intervention)	16
Primary outcome variable	The International Hip Outcome Tool–33	x	x	x	x
Secondary outcome variables	Motor cortex excitability	x		x	
	Voluntary activation	x		x	
	Conditioned pain modulation	x		x	
	EuroQol 5 dimensions	x	x	x	x
Other variables	Demographics	x		x	
	Kellgren Lawrence (KL) Score	x			
	Scoring hip osteoarthritis with MRI (SHOMRI)	x			
	Flare-Osteoarthritis questionnaire	X	X	X	X
	Sit to stand pain	x		x	
	Muscle strength	x		x	
	Medication	x	x	x	x
	Physical activity	x	x	x	x
	Work status	x	x	x	x
	Dietary intake	x			
	Lifestyle Measures	x			
	Psychological Measures	x			
	Adherence	Continual			
	Adverse events	Continual			

#### 2.10.1 Primary outcome variable

##### 2.10.1.1 Pain, Disability and QoL

The International Hip Outcome Tool–33 (iHOT-33) is a valid and reliable tool to assess pain, disability and quality of life in people with hip OA, and recommended by the 2018 Zurich consensus [17]. The iHOT-33 has a range of scores from 0 to 100 and a minimal important change (MIC) of 12.5 points [18].

#### 2.10.2 Secondary outcome variables

##### 2.10.2.1 Motor cortex excitability

Transcranial magnetic stimulation (TMS) is a valid method that elicits quadriceps motor responses, assessing cortex excitability/inhibition including: motor evoked potential; resting/active motor threshold; short-interval intracortical inhibition; intracortical facilitation; silent period [16].

##### 2.10.2.2 Voluntary Activation

Voluntary activation of the quadriceps will be assessed by generating an electrically evoked muscle twitch, using supramaximal femoral nerve stimulation, which will be superimposed onto a maximal voluntary knee extension force (MVC) and then performed with the muscles at rest. Voluntary activation (%) will be calculated as (1 ​− ​superimposed twitch amplitude/resting twitch amplitude) × 100. Participants will be in a seated position and performing a maximal knee extension in our custom-built chair. The participant will be familiar with the MVC procedure from their peripheral muscle strength testing with trials structured in an identical manner. Stimulation intensity will be identified by gradually increasing intensity until the resting knee extensor twitch response is maximal [19].

##### 2.10.2.3 Conditioned pain modulation

Pressure pain thresholds (kiloPascals, kPa) of the hip, over the superior border of the greater trochanter, and the ipsilateral lateral epicondyle of the elbow, assessed using an algometer, will be used as the test stimulus and icy cold water (∼5 ​°C) immersion of the hand (contralateral hand to the most painful hip) for 3 ​min as conditioning stimulus [20]. The CPM effect will be calculated as the absolute (kPa) and relative (percentage) pre-post ice immersion pressure pain threshold change [10,21].

##### 2.10.2.4 Health-related QoL

The EuroQol 5 dimensions (EQ-5D-5L) is a valid and reliable tool to quantify health-related QoL [22].

#### 2.10.3 Other variables

##### 2.10.3.1 Demographic data

Age (years), sex (male; female; intersex), race (White; Black or African American; American Indian or Alaska Native; Asian; Native Hawaiian or Other Pacific Islander), height (cm), weight (kg), duration of hip pain (weeks), education level (Less than high school; High school graduate; Bachelor's degree; Master's degree; Doctoral degree; Professional degree), occupation, employment status (Full time; Part time; Home maker; Student; Retired; Other) and household income (Less than 30,000 AUD; 30,000–49,999 AUD; 50,000–79,999 AUD; 80,000–99,999 AUD; 100,000–149,999 AUD; 150,000–199,999 AUD; Greater than 200,000 AUD; Decline to answer).

##### 2.10.3.2 Severity of radiographic OA

Assessed *via* x-ray using the Kellgren Lawrence (KL) scoring system and *via* MRI using the Scoring Hip Osteoarthritis with MRI (SHOMRI), reported by a single radiologist from the partner radiology clinic. The KL score will be an ordinal score between 0 and 4, with scores of 2 or greater representing radiographic osteoarthritis [23]. The SHOMRI score will include the assessment of eight core hip joint features (articular cartilage lesions, bone marrow oedema, subchondral cysts, labral abnormalities, paralabral cysts, intra-articular loose bodies, joint effusion, and ligamentum teres abnormalities) with an ordinal score assigned to each feature [24].

##### 2.10.3.3 Flare-OA Questionnaire

The Flare-OA Questionnaire is a valid and reliable tool to evaluate the presence and severity of any OA flare [25].

##### 2.10.3.4 Pain with sit-to-stand

The 11-point numerical rating scale of pain (NRS-P) will be used to determine pain from a functional activity that is provocative in hip OA. The squat depth will be standardised to a set height (standard 20-inch gym box) and patients will be provided standardised instructions to report the worst NRS-P from five repetitions.

##### 2.10.3.5 Hip and knee muscle strength

Maximal isometric hip abduction, hip adduction, hip flexion, hip extension and knee flexion strength [26] will be measured using a hand-held dynamometer. Participants will perform several submaximal tests to familiarise themselves with each action before completing three maximal voluntary contraction (MVC) tests, pushing for 3 ​s per trial and with a minimum of 30-s rest between trials. We will record peak force within each trial to calculate the average force, the maximal force and the co-efficient of variation of the three trials. The distance from the greater trochanter to the dynamometer (hip measures) and patellar apex to dynamometer (knee measures) will be measured to allow calculation of torque. An identical approach will be used for knee extension, however this will be performed in a custom-built chair with the ankle secured within a splint connected to a force transducer, prior to TMS, as quadriceps maximal contraction strength will determine TMS/voluntary activation parameters. Participants will be positioned to ensure consistent, controlled contractions during assessment.

##### 2.10.3.6 Medications

All medications (pain and general medications) will be recorded.

##### 2.10.3.7 Physical activity level

The World Health Organisation Global Physical Activity Questionnaire (GPAQ) [27] will be used to quantify physical activity levels.

##### 2.10.3.8 Lifestyle measures

Smoking status (Yes/No) and alcohol intake (drinks per week) will also be recorded prior to physical assessment.

##### 2.10.3.9 Psychological measures

The generalised anxiety disorder (GAD-7) [28], patient health questionnaire (PHQ-9) [29] and depression, anxiety and stress scale (DASS) [30] will be completed. The 11-item TAMPA Scale of Kinesiophobia will be used to assess the extent of fear of movement in participants [31].

##### 2.10.3.10 Exercise and consultation adherence

Exercise adherence will be recorded as the number of consultations attended, which will be extracted from the clinical records at the conclusion of the intervention window.

##### 2.10.3.11 Adverse events

Adverse events will be directly reported to study staff and recorded with date, time and description of incident/event.

### 2.11 Adverse events

tDCS has an excellent safety profile [32]. tDCS has few adverse events when applied with stimulation parameters in line with published guidelines. Adverse events from tDCS are rare or mild, with mild tingling/itching of the scalp most common [6,7,32].

### 2.12 Team and participant communication

Once enrolled, all participants will be added to their own individual WhatsApp chat group with the clinical trial coordinator, supervising physiotherapist, and Dr Murphy to allow for open dialogue.

### 2.13 Power calculation

Using G∗Power v3: *n* ​= ​64 is sufficient to detect between-group differences if greater than the iHOT-33 MIC ​= ​12.5 points (*d* ​= ​0.71) [19], *α* ​= ​0.05, 1-*β* ​= ​0.80. Thus, we will recruit *n* ​= ​78 hip OA participants (*n* ​= ​39 per arm) accounting for an expected portion being lost to follow-up (drop-out ∼20% expected from pilot studies in knee OA [6]).

### 2.14 Timeline

**Fig. 1:**
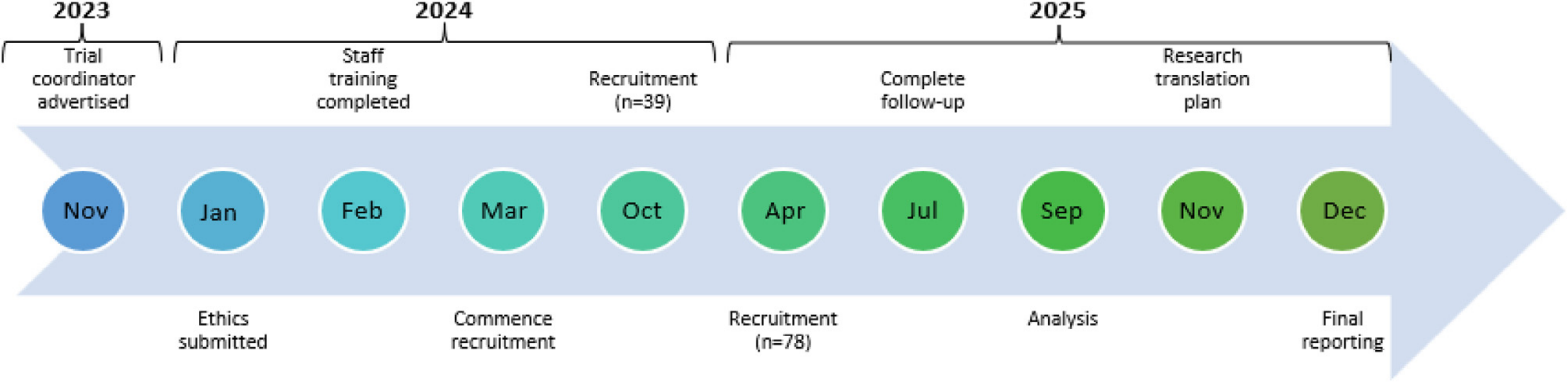
STIM hips timeline.

Our estimated timeline is presented in Fig. 1.

### 2.15 Data management

Data will be managed in accordance with existing National Health and Medical Research Council of Australia (NHMRC)/Edith Cowan University policies.

### 2.16 Risk management

Risk management for this RCT will be executed using the comprehensive Edith Cowan University risk management framework and reporting system (RiskWare). This system must be completed for ethical approval and involves logging details of actions such as completion of TMS or tDCS training for the clinical trial coordinator or engineering maintenance of TMS equipment. RiskWare is managed by the university risk management team who will support this research project.

## 3 Analysis

### 3.1 Statistical analysis

All data will be described at baseline and across each time point. Data distributions will be examined for each outcome using Shapiro Wilk test and guide preliminary statistical between group test selections. Repeated mixed effects models will determine between-group differences for the primary outcome (iHOT-33), accounting for relevant confounders (i.e., age; sex; BMI; radiographic severity) with relevant model assumptions examined. Secondary analysis will determine between-group differences for the other outcomes of interest (cortex excitability and CPM). Results will be reported according to EQUATOR Network reporting guidelines which include SAMPL and CONSORT. Statistical analysis will be performed by a blinded biostatistician from a separate institute.

### 3.2 Economic analysis

Participants will have no out-of-pocket costs. However, usual financial costs ($AUD) associated with tDCS usage and supervised physiotherapy (if participants had not been included in the trial and had to pay market rates) will be recorded using market average. Furthermore, market average costs from reported medications will be included. A subsequent economic evaluation with the healthcare system as the agent subject to costs, will estimate the incremental cost associated with the intervention per quality-adjusted life year using the EQ-5D-5L.

### 3.3 Process evaluation

Implementation appraisal will occur following data collection (e.g., after the 16-week follow-up). STIM HIPS participants will be invited to small focus group interviews until saturation is reached. By exploring participant tDCS experiences, perceived barriers/facilitators to implementation will be identified. A qualitative descriptive study approach will be used to focus research questions directly on participant experience, as in our previous research [33,34]. QSR NVIVO will be used for coding and analysing the qualitative data using a deductive thematic approach. Results will be reported according to the consolidated criteria for reporting qualitative research (COREQ) [35].

## 4 Implications

Given the massive worldwide prevalence, and that one-in-eleven Australians suffer from OA [36], it has been recognised within the Australian national OA strategy that immediate action is needed to reduce the personal, workforce and healthcare burden of this disease [37]. However, this is not a simple task, and OA is just one part of the picture. Australians with OA are twice as likely to report having ‘poor health’ versus those without [36]. Furthermore, these Australians typically present with several chronic health conditions (e.g., lower back pain, mental health complaints, cardiac disease or diabetes) [36] associated with decreased function and poor QoL. Therefore, even with the invention of a magic bullet to resolve OA pain, the impaired function and poor QoL would be unlikely to meaningfully change. Thus, interventions must be prioritised that can address pain, but also function and quality of life. An abundance of research details the benefits to pain, function and QoL from regular physical activity and good nutrition in conditions such as OA, cardiac disease and diabetes [38]. However, people with hip/knee OA have a significantly reduced level of physical activity compared to the general population [39]. These reduced levels of physical activity are, in part, due to fear that increased physical activity will make OA pain worse [40]. Unfortunately, this is a maladaptive response and further reductions in physical activity only serve to increase symptoms and reduce QoL.

**Fig. 2:**
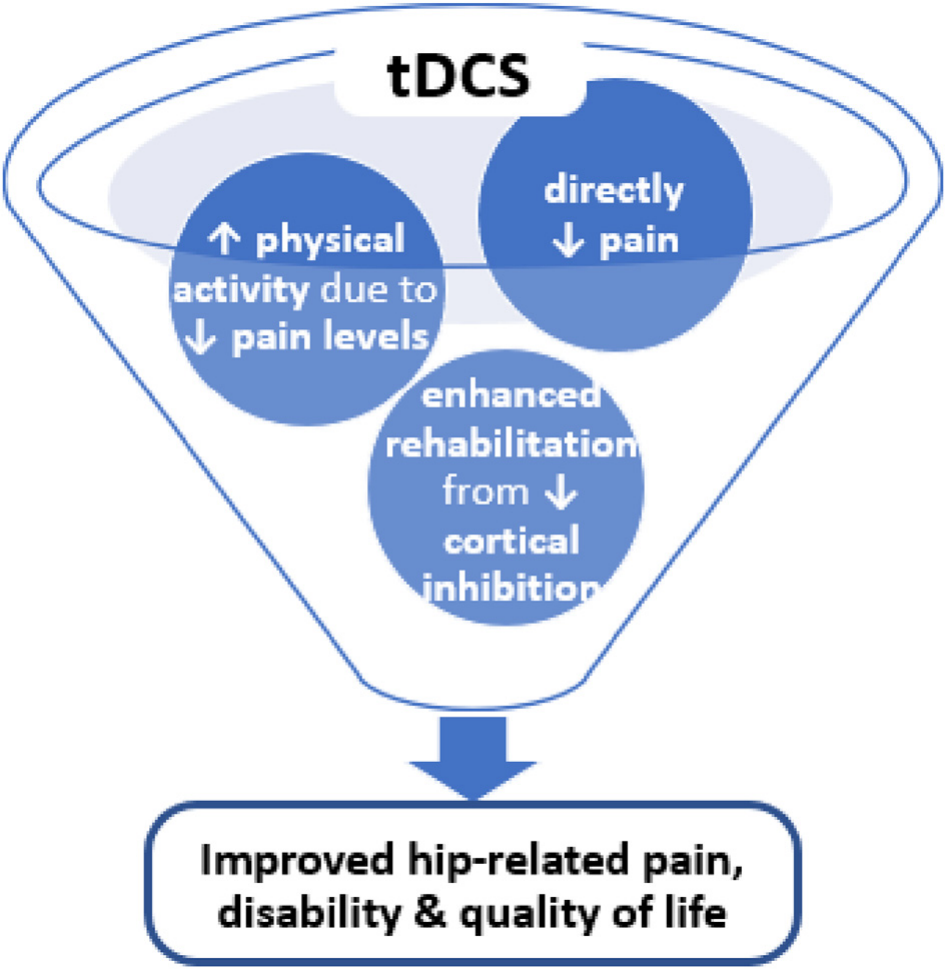
Visualisation of the effect of tDCS.

The tDCS intervention described in this study protocol aims to address pain, function, and QoL in people with hip OA. tDCS has been demonstrated to reduce pain in people with OA [6,7]. Capitalising on the potential reduction of pain will also enable people with OA to increase their physical activity levels, providing the benefits of tDCS by relieving pain, and the associated benefits of increased physical activity. Furthermore, muscle strength can be increased during tDCS through altered cortical activity. As this should allow exercise with higher loads, enhanced muscular adaptation secondary to tDCS application should translate to greater strength improvement with training in people with hip OA. For example, if a leg press weight of 30 ​kg was required to improve muscle mass (hypertrophy) in an individual but voluntary activation deficits and cortical inhibition meant they were only able to provide 25 ​kg of force, they would be highly unlikely to increase muscle mass, which is a primary aim of rehabilitation. The adjunct use of tDCS is hypothesised to be able to overcome this barrier, and when applied, allow the person to produce 30 ​kg of force their muscle is more than capable of generating, but their brain was stopping them from accessing. Thus, tDCS will improve hip-related pain, disability and QoL *via* three mechanisms (Fig. 2): 1) directly reducing pain through tDCS application, 2) increased physical activity levels due to reduced pain from tDCS application, and 3) enhanced outcomes from rehabilitation due to additional muscular adaptation by using tDCS to reduce cortical inhibition.

Finally, the prevalence of hip OA is increasing at a significantly faster rate than knee OA [2] but the majority of the research is focussed on interventions for knee OA. Whilst that may not seem important, equity concerns exist as women have a far higher burden of hip OA than men [41]. However, this is unsurprising as women, including research into musculoskeletal conditions and treatment affecting women, have traditionally been substantially under-represented in sport science, physiotherapy and medical research [42]. The STIM Hips trial will address these research gaps by investigating a novel intervention to address pain, function and QoL in people with hip OA.

## Funding information

This clinical trial is funded by a Raine Medical Research Foundation Priming Grant (G1006856), awarded to Dr Myles C Murphy.

## Declaration of competing interest

The authors declare no competing interests.
